# Ischemia Possibly Associated with High Degree Atrioventricular Block

**DOI:** 10.1155/2023/6676757

**Published:** 2023-08-19

**Authors:** Dimitrios Siamkouris, Elmar Offers, Marc Schloesser, Gjoko Ilieski, Stergios Tzikas

**Affiliations:** ^1^Department of Cardiology, Dreifaltigkeits Hospital Lippstadt, Germany, Academic Teaching Hospital of Westfaelische Wilhelms University Muenster, Muenster, Germany; ^2^3rd Department of Cardiology, Ippokrateio General Hospital, Aristotle University of Thessaloniki, Thessaloniki, Greece

## Abstract

Conduction restoration in second- and third-degree atrioventricular (AV) block after revascularization in acute coronary syndrome (ACS) setting is well established, however this is not the case in non-ACS setting. We present a case of a patient referred for permanent pacemaker implantation, due to high degree AV block (HAVB), who restored his conduction possibly due to targeted revascularization. Thus, this case sheds insight in the reversibility potential of HAVB after revascularization in non-ACS patients, which although signified in current literature, is still far from being a recommendation, due to lack of robust scientific confirmation.

## 1. Introduction

The perfusion of the atrioventricular (AV) conduction system is normally sustained through the AV node artery and the proximal septal perforators. Conduction restoration in high degree AV block (HAVB), which encloses second- and third-degree AV block, after revascularization in acute coronary syndrome (ACS) setting is well established, however this is not the case in non-ACS setting [1].

## 2. Case Presentation

A 60-year-old male patient was referred for permanent pacemaker implantation, after being diagnosed with third-degree AV block. The patient reported an episode of syncope in supine position, without prodrome and complained of dizziness and easy fatigability ever since. He also acknowledged suffering angina pectoris episodes during physical activity in the last 5 months.

He had been diagnosed with arterial hypertension 5 years ago, and his medication comprised Ramipril 10 mg od, Amlodipine 5 mg od. and Hydrochlorothiazide 25 mg od. Drug and tobacco abuse have been refused. Besides his bradycardia the clinical examination showed no further abnormality.

**Figure 1:**
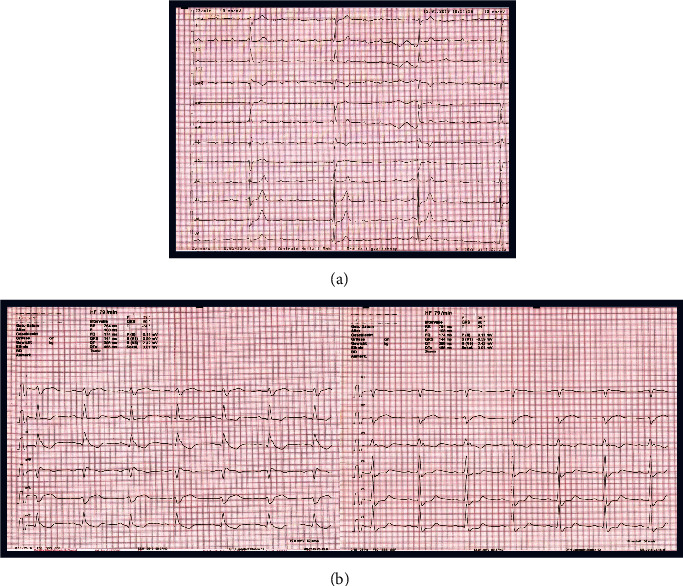
(a) ECG of the patient at presentation. Third degree AV Block with a ventricular escape rhythm and a rate of 22/min. (b) ECG of the patient after percutaneous coronary intervention of the proximal LAD at the site of origin of a large first septal and smaller second and third septal perforator. Sinus rhythm with 1 : 1 AV conduction, right bundle branch block (RBBB) and left posterior fascicular block.

**Figure 2:**
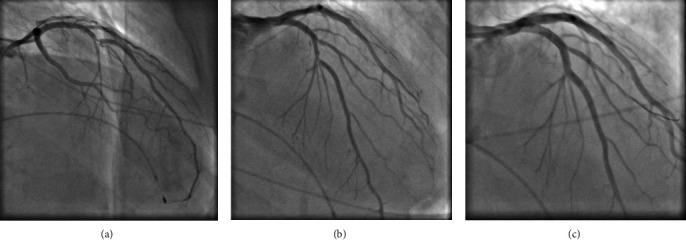
(a) and (b) Different angulations of the LAD stenosis at the site of origin of a large first and a smaller second and third septal perforator (arrow pointing to the lesion). (c) Result after predilatation, stent deployment and POT proximal to the first septal perforator.

The ECG revealed a third-degree AV block with a ventricular escape rhythm (Figure 1(a)). A bed side transthoracic echocardiography showed a normal left ventricular ejection fraction without any significant structural pathologies. Laboratory testing showed no abnormality. In consideration of the unclear cause of the HAVB and the reported angina, we performed a coronary angiography to rule out an ischemic cause of HAVB. A severe stenosis of the proximal-to-mid LAD at the site of origin of a large first septal perforator (FSP) and a smaller second and third septal branch was revealed (Figures 2(a) and 2(b), Video 1, Video 2). Upon revascularization (Figure 2(c), Video 3), performed under temporary pacing, the patient surprisingly, reverted to 1 : 1 AV conduction, with a right bundle branch block (RBBB) and a left posterior fascicular block present (Figure 1(b)). Therefore, permanent pacemaker implantation was deferred and after 5 days of continuous ECG monitoring with persisting 1 : 1 AV conduction we decided to implant a loop recorder (ILR) and discharged the patient. Due to a single transient five seconds lasting episode of HAVB one month after discharge the patient indeed received in a separate in-patient hospital stay a permanent pacemaker. However, follow up pacemaker interrogation in 2 weeks and 2 months revealed no measurable right ventricular pacing (<1%).

## 3. Discussion

The present case sheds insight in the reversibility potential of HAVB after targeted revascularization in non-ACS patients, which although signified in current literature, is still far from being a recommendation, due to lack of robust scientific confirmation.

A small number of case-reports already indicate the relationship between revascularization and AV conduction restoration [2–4]. However, this relationship was not present in a case series of patients with delayed and partially incomplete coronary artery bypass graft surgery (CABG) revascularization [5]. The importance of the revascularization quality both measured through its targeting and completeness was apparent in another small study [6].

In our case pacemaker was finally inevitable, however resolution of complete AV block upon targeted revascularization of a lesion interfering with the perfusion of the AV conduction system, in a patient with persistent HAVB for many hours and subsequent lack of measurable right ventricular stimulation ever since, allows the hypothesis of a possible association to the implemented coronary intervention. Ischemia workup however, remains not a routine diagnostic step for patients with isolated HAVB, unless coronary artery disease is highly suspected [1].

Furthermore, this case demonstrates that even if complete recovery and avoidance of a permanent pacemaker implantation does not occur, the reduction of the need for right ventricular stimulation, has further beneficial effects to be considered. Among them, the reduced risk of pacing-induced cardiomyopathy [7], increased battery longevity and reduced risk of atrial fibrillation [8].

All the above point the need for better prediction of conduction responsiveness to revascularization. However, there is also a gap in evidence regarding the subsequent management of patients with reversal of HAVB after revascularization. ILR implantation seems a reasonable alternative to elucidate permanency of normal AV conduction, especially when taking into consideration the low negative predictive value of an electrophysiological study [1].

Overall, the present case report adds to prior publications in acknowledging the potential of reversibility of a HAVB after targeted revascularization, addresses the question of durability of HAVB resolution and suggests a possible proceeding in the clinical scenario of an initially complete resolution of HAVB, using an ILR. This proceeding could be considered as a novelty in the comprehensive treatment of such selected patients, as no recommendations exist. Finally, this case demonstrates that even non-persistent resolution of HAVB could enclose beneficial effects. However, it is necessary to emphasize that due to lack of robust scientific evidence, diagnostic investigation of ischemia cannot be, as of today, recommended in the diagnostic workup in patients with HAVB.

## Supplementary Materials

Supplementary Materials Video 1: Angiogram of the left coronary artery in right anterior oblique cranial projection depicting the severe stenosis of the proximal-to-mid LAD at the site of origin of a large first septal perforator branch. Video 2: Angiogram of the left coronary artery in anteroposterior cranial projection depicting the severe stenosis of the proximal-to-mid LAD at the site of origin of a large first septal perforator branch. Video 3: Angiogram of the left coronary artery in anteroposterior cranial projection depicting the the result after angioplasty of the proximal-to-mid LAD.
