# Supplementary material for: Ischemia Possibly Associated with High Degree Atrioventricular Block
Source: Case Rep Cardiol. 2023 Aug 19;2023:6676757. doi: 10.1155/2023/6676757 (PMC10460276; doi:10.1155/2023/6676757)
Supplement: Supplementary Materials — Video 1: Angiogram of the left coronary artery in right anterior oblique cranial projection depicting the severe stenosis of the proximal-to-mid LAD at the site of origin of a large first septal perforator branch. Video 2: Angiogram of the left coronary artery in anteroposterior cranial projection depicting the severe stenosis of the proximal-to-mid LAD at the site of origin of a large first septal perforator branch. Video 3: Angiogram of the left coronary artery in anteroposterior cranial projection depicting the the result after angioplasty of the proximal-to-mid LAD. [file 6676757.f1.docx]

Links for Videos:

<https://drive.google.com/file/d/1dMw0Ht5ny0_MGUi4RVwwGu626xTP_rpj/view?usp=share_link>

<https://drive.google.com/file/d/1102HBwDItFTZrj9DjkouSW-VcFxlgCJZ/view?usp=share_link>

<https://drive.google.com/file/d/1GmWXJYLmDGvOILnd7Vdbt4nsPIwcbTto/view?usp=share_link>
